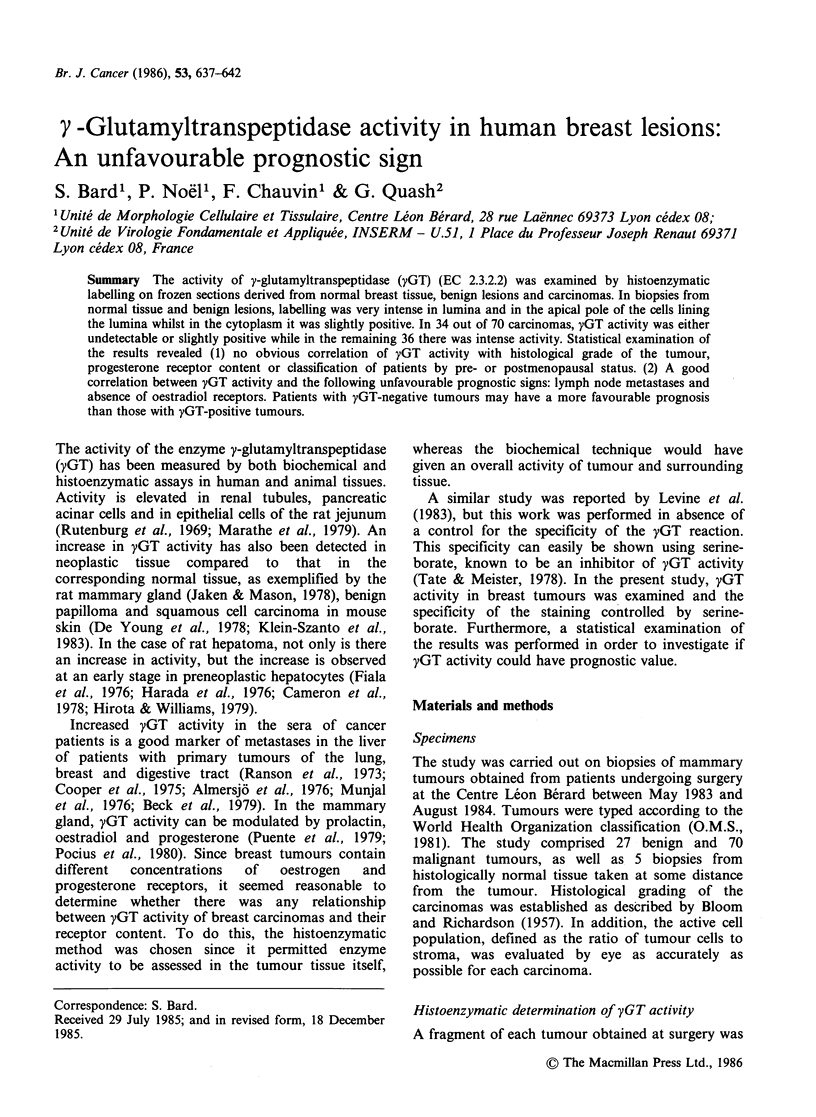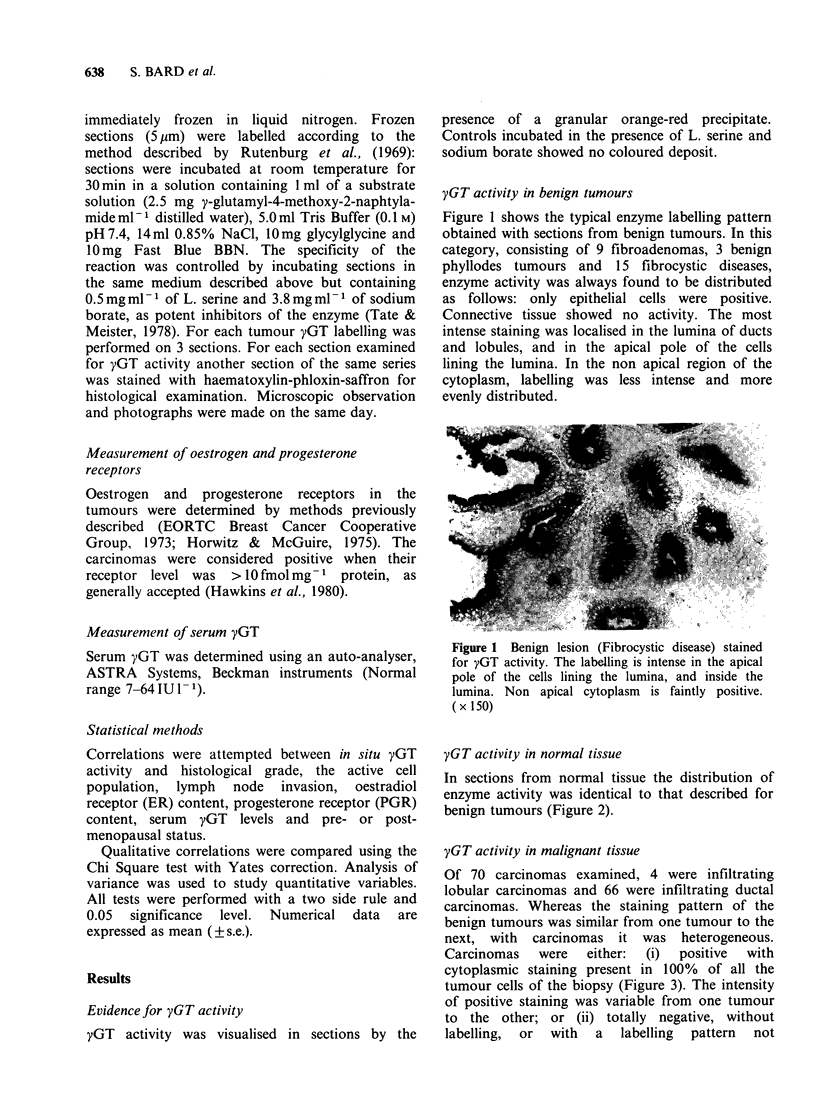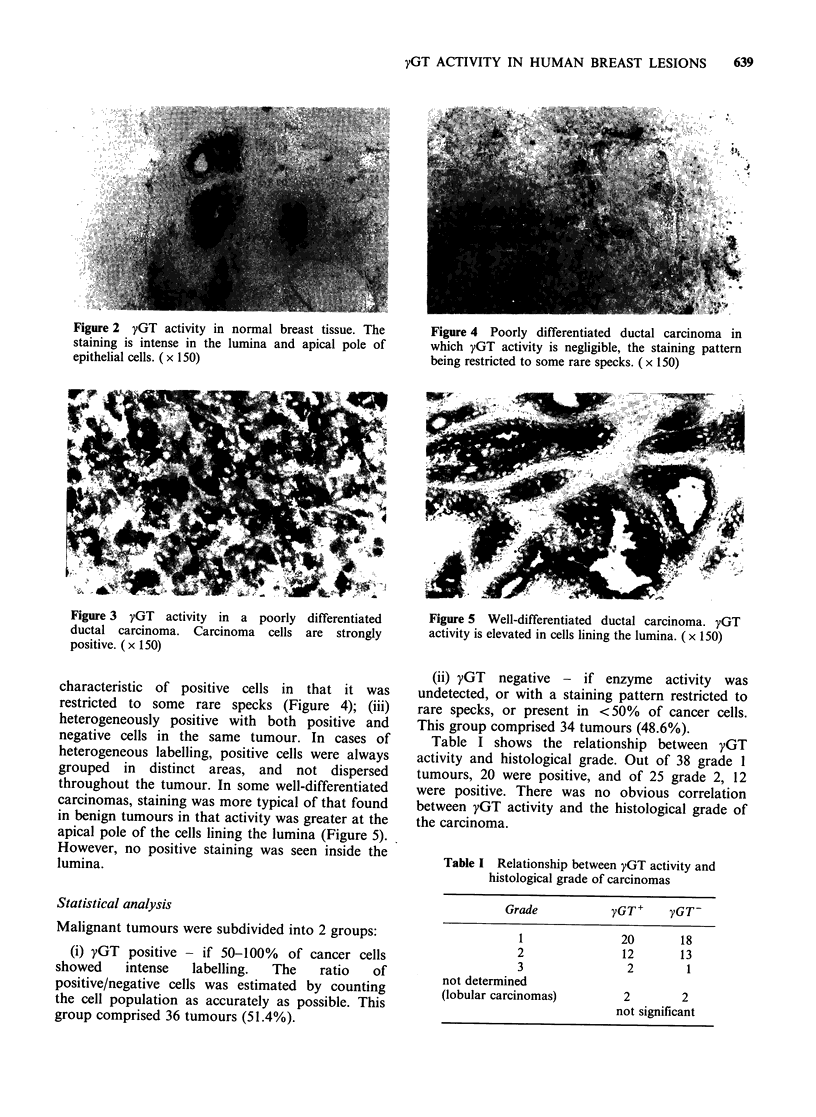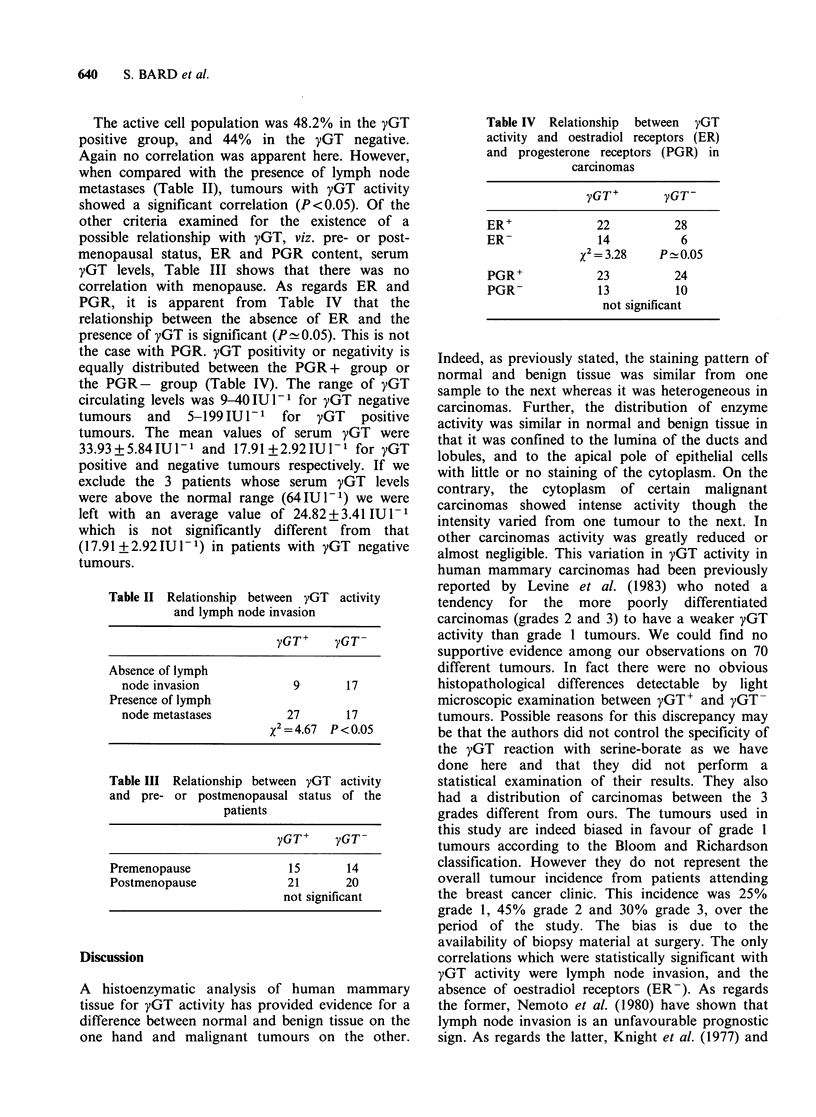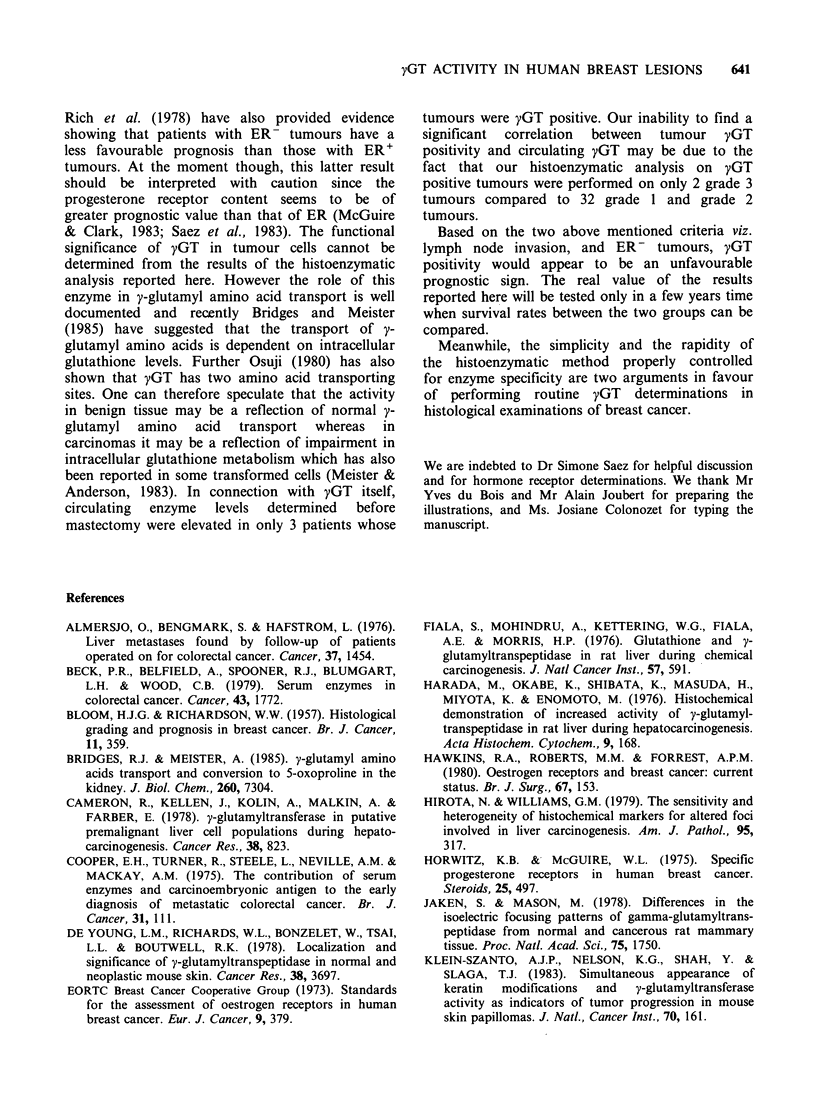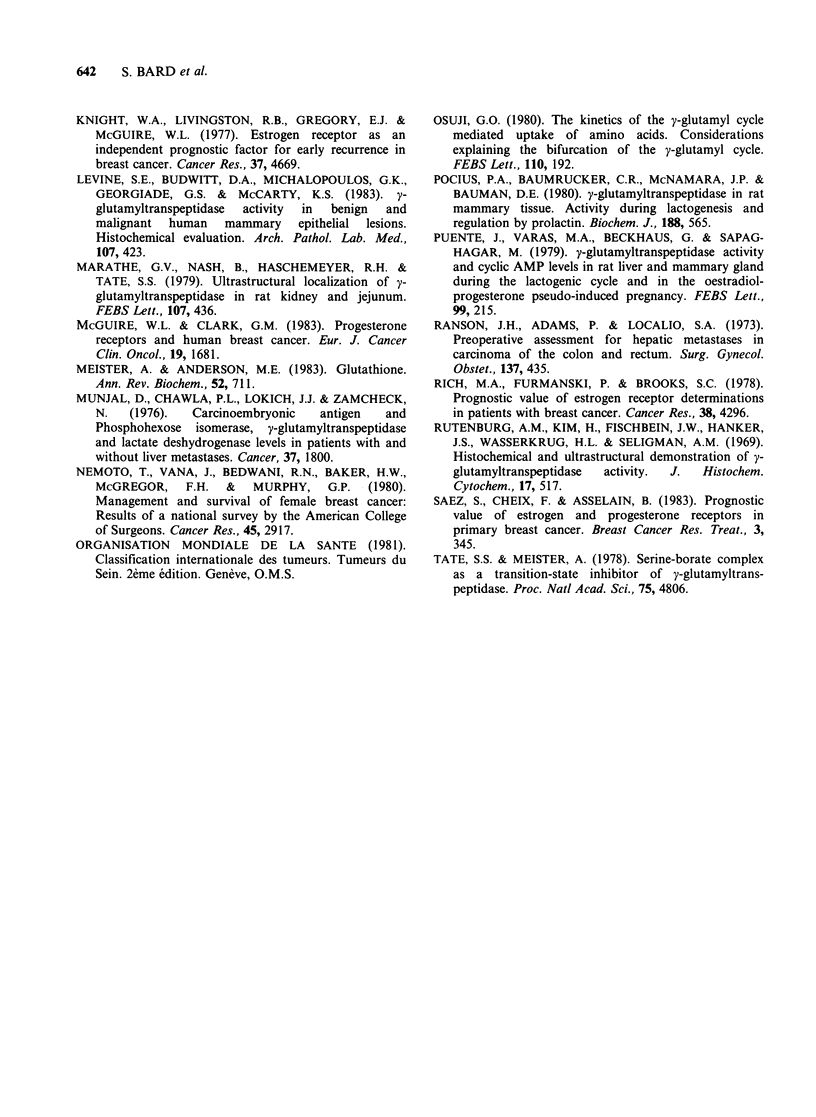# gamma-Glutamyltranspeptidase activity in human breast lesions: an unfavourable prognostic sign.

**DOI:** 10.1038/bjc.1986.107

**Published:** 1986-05

**Authors:** S. Bard, P. Noël, F. Chauvin, G. Quash

## Abstract

**Images:**


*Br. J. Cancer* (1986), **53**, 637–642

### γ-Glutamyltranspeptidase activity in human breast lesions: An unfavourable prognostic sign

S. Bard[1], P. Noël[1], F. Chauvin[1] & G. Quash[2]

[1]*Unité de Morphologie Cellulaire et Tissulaire, Centre Léon Bérard, 28 rue Laënnec 69373 Lyon cédex 08;* [2]*Unité de Virologie Fondamentale et Appliquée, INSERM – U.51, 1 Place du Professeur Joseph Renaut 69371 Lyon cédex 08, France*

**Summary** The activity of γ-glutamyltranspeptidase (γGT) (EC 2.3.2.2) was examined by histoenzymatic labelling on frozen sections derived from normal breast tissue, benign lesions and carcinomas. In biopsies from normal tissue and benign lesions, labelling was very intense in lumina and in the apical pole of the cells lining the lumina whilst in the cytoplasm it was slightly positive. In 34 out of 70 carcinomas, γGT activity was either undetectable or slightly positive while in the remaining 36 there was intense activity. Statistical examination of the results revealed (1) no obvious correlation of γGT activity with histological grade of the tumour, progesterone receptor content or classification of patients by pre- or postmenopausal status. (2) A good correlation between γGT activity and the following unfavourable prognostic signs: lymph node metastases and absence of oestradiol receptors. Patients with γGT-negative tumours may have a more favourable prognosis than those with γGT-positive tumours.

The activity of the enzyme γ-glutamyltranspeptidase (γGT) has been measured by both biochemical and histoenzymatic assays in human and animal tissues. Activity is elevated in renal tubules, pancreatic acinar cells and in epithelial cells of the rat jejunum (Rutenburg *et al.*, 1969; Marathe *et al.*, 1979). An increase in γGT activity has also been detected in neoplastic tissue compared to that in the corresponding normal tissue, as exemplified by the rat mammary gland (Jaken & Mason, 1978), benign papilloma and squamous cell carcinoma in mouse skin (De Young *et al.*, 1978; Klein-Szanto *et al.*, 1983). In the case of rat hepatoma, not only is there an increase in activity, but the increase is observed at an early stage in preneoplastic hepatocytes (Fiala *et al.*, 1976; Harada *et al.*, 1976; Cameron *et al.*, 1978; Hirota & Williams, 1979).

Increased γGT activity in the sera of cancer patients is a good marker of metastases in the liver of patients with primary tumours of the lung, breast and digestive tract (Ranson *et al.*, 1973; Cooper *et al.*, 1975; Almersjö *et al.*, 1976; Munjal *et al.*, 1976; Beck *et al.*, 1979). In the mammary gland, γGT activity can be modulated by prolactin, oestradiol and progesterone (Puente *et al.*, 1979; Pocius *et al.*, 1980). Since breast tumours contain different concentrations of oestrogen and progesterone receptors, it seemed reasonable to determine whether there was any relationship between γGT activity of breast carcinomas and their receptor content. To do this, the histoenzymatic method was chosen since it permitted enzyme activity to be assessed in the tumour tissue itself, whereas the biochemical technique would have given an overall activity of tumour and surrounding tissue.

A similar study was reported by Levine *et al.* (1983), but this work was performed in absence of a control for the specificity of the γGT reaction. This specificity can easily be shown using serine-borate, known to be an inhibitor of γGT activity (Tate & Meister, 1978). In the present study, γGT activity in breast tumours was examined and the specificity of the staining controlled by serine-borate. Furthermore, a statistical examination of the results was performed in order to investigate if γGT activity could have prognostic value.

### Materials and methods

### *Specimens*

The study was carried out on biopsies of mammary tumours obtained from patients undergoing surgery at the Centre Léon Bérard between May 1983 and August 1984. Tumours were typed according to the World Health Organization classification (O.M.S., 1981). The study comprised 27 benign and 70 malignant tumours, as well as 5 biopsies from histologically normal tissue taken at some distance from the tumour. Histological grading of the carcinomas was established as described by Bloom and Richardson (1957). In addition, the active cell population, defined as the ratio of tumour cells to stroma, was evaluated by eye as accurately as possible for each carcinoma.

### *Histoenzymatic determination of γGT activity*

A fragment of each tumour obtained at surgery was

Correspondence: S. Bard.
Received 29 July 1985; and in revised form, 18 December 1985.

© The Macmillan Press Ltd., 1986

immediately frozen in liquid nitrogen. Frozen sections (5 μm) were labelled according to the method described by Rutenburg *et al.*, (1969): sections were incubated at room temperature for 30 min in a solution containing 1 ml of a substrate solution (2.5 mg γ-glutamyl-4-methoxy-2-naphtylamide $ml^{-1}$ distilled water), 5.0 ml Tris Buffer (0.1 M) pH 7.4, 14 ml 0.85% NaCl, 10 mg glycylglycine and 10 mg Fast Blue BBN. The specificity of the reaction was controlled by incubating sections in the same medium described above but containing 0.5 mg $ml^{-1}$ of L. serine and 3.8 mg $ml^{-1}$ of sodium borate, as potent inhibitors of the enzyme (Tate & Meister, 1978). For each tumour γGT labelling was performed on 3 sections. For each section examined for γGT activity another section of the same series was stained with haematoxylin-phloxin-saffron for histological examination. Microscopic observation and photographs were made on the same day.

*Measurement of oestrogen and progesterone receptors*

Oestrogen and progesterone receptors in the tumours were determined by methods previously described (EORTC Breast Cancer Cooperative Group, 1973; Horwitz & McGuire, 1975). The carcinomas were considered positive when their receptor level was >10 fmol $mg^{-1}$ protein, as generally accepted (Hawkins *et al.*, 1980).

*Measurement of serum γGT*

Serum γGT was determined using an auto-analyser, ASTRA Systems, Beckman instruments (Normal range 7–64 IU $l^{-1}$).

*Statistical methods*

Correlations were attempted between *in situ* γGT activity and histological grade, the active cell population, lymph node invasion, oestradiol receptor (ER) content, progesterone receptor (PGR) content, serum γGT levels and pre- or post-menopausal status.

Qualitative correlations were compared using the Chi Square test with Yates correction. Analysis of variance was used to study quantitative variables. All tests were performed with a two side rule and 0.05 significance level. Numerical data are expressed as mean (±s.e.).

### Results

*Evidence for γGT activity*

γGT activity was visualised in sections by the presence of a granular orange-red precipitate. Controls incubated in the presence of L. serine and sodium borate showed no coloured deposit.

*γGT activity in benign tumours*

Figure 1 shows the typical enzyme labelling pattern obtained with sections from benign tumours. In this category, consisting of 9 fibroadenomas, 3 benign phyllodes tumours and 15 fibrocystic diseases, enzyme activity was always found to be distributed as follows: only epithelial cells were positive. Connective tissue showed no activity. The most intense staining was localised in the lumina of ducts and lobules, and in the apical pole of the cells lining the lumina. In the non apical region of the cytoplasm, labelling was less intense and more evenly distributed.

**Figure 1** Benign lesion (Fibrocystic disease) stained for γGT activity. The labelling is intense in the apical pole of the cells lining the lumina, and inside the lumina. Non apical cytoplasm is faintly positive. (×150)

*γGT activity in normal tissue*

In sections from normal tissue the distribution of enzyme activity was identical to that described for benign tumours (Figure 2).

*γGT activity in malignant tissue*

Of 70 carcinomas examined, 4 were infiltrating lobular carcinomas and 66 were infiltrating ductal carcinomas. Whereas the staining pattern of the benign tumours was similar from one tumour to the next, with carcinomas it was heterogeneous. Carcinomas were either: (i) positive with cytoplasmic staining present in 100% of all the tumour cells of the biopsy (Figure 3). The intensity of positive staining was variable from one tumour to the other; or (ii) totally negative, without labelling, or with a labelling pattern not

**Figure 2** γGT activity in normal breast tissue. The staining is intense in the lumina and apical pole of epithelial cells. ( × 150)

**Figure 3** γGT activity in a poorly differentiated ductal carcinoma. Carcinoma cells are strongly positive. ( × 150)

**Figure 4** Poorly differentiated ductal carcinoma in which γGT activity is negligible, the staining pattern being restricted to some rare specks. ( × 150)

**Figure 5** Well-differentiated ductal carcinoma. γGT activity is elevated in cells lining the lumina. ( × 150)

characteristic of positive cells in that it was restricted to some rare specks (Figure 4); (iii) heterogeneously positive with both positive and negative cells in the same tumour. In cases of heterogeneous labelling, positive cells were always grouped in distinct areas, and not dispersed throughout the tumour. In some well-differentiated carcinomas, staining was more typical of that found in benign tumours in that activity was greater at the apical pole of the cells lining the lumina (Figure 5). However, no positive staining was seen inside the lumina.

*Statistical analysis*

Malignant tumours were subdivided into 2 groups:

(i) γGT positive – if 50–100% of cancer cells showed intense labelling. The ratio of positive/negative cells was estimated by counting the cell population as accurately as possible. This group comprised 36 tumours (51.4%).

(ii) γGT negative – if enzyme activity was undetected, or with a staining pattern restricted to rare specks, or present in <50% of cancer cells. This group comprised 34 tumours (48.6%).

Table I shows the relationship between γGT activity and histological grade. Out of 38 grade 1 tumours, 20 were positive, and of 25 grade 2, 12 were positive. There was no obvious correlation between γGT activity and the histological grade of the carcinoma.

**Table I** Relationship between γGT activity and histological grade of carcinomas

| *Grade* | $\gamma GT^+$ | $\gamma GT^-$ |
|---|---|---|
| 1 | 20 | 18 |
| 2 | 12 | 13 |
| 3 | 2 | 1 |
| not determined (lobular carcinomas) | 2 | 2 |
| | not significant | |

The active cell population was 48.2% in the $\gamma$GT positive group, and 44% in the $\gamma$GT negative. Again no correlation was apparent here. However, when compared with the presence of lymph node metastases (Table II), tumours with $\gamma$GT activity showed a significant correlation ($P<0.05$). Of the other criteria examined for the existence of a possible relationship with $\gamma$GT, *viz.* pre- or postmenopausal status, ER and PGR content, serum $\gamma$GT levels, Table III shows that there was no correlation with menopause. As regards ER and PGR, it is apparent from Table IV that the relationship between the absence of ER and the presence of $\gamma$GT is significant ($P\simeq 0.05$). This is not the case with PGR. $\gamma$GT positivity or negativity is equally distributed between the PGR+ group or the PGR− group (Table IV). The range of $\gamma$GT circulating levels was 9–40 IU $l^{-1}$ for $\gamma$GT negative tumours and 5–199 IU $l^{-1}$ for $\gamma$GT positive tumours. The mean values of serum $\gamma$GT were $33.93 \pm 5.84$ IU $l^{-1}$ and $17.91 \pm 2.92$ IU $l^{-1}$ for $\gamma$GT positive and negative tumours respectively. If we exclude the 3 patients whose serum $\gamma$GT levels were above the normal range (64 IU $l^{-1}$) we were left with an average value of $24.82 \pm 3.41$ IU $l^{-1}$ which is not significantly different from that ($17.91 \pm 2.92$ IU $l^{-1}$) in patients with $\gamma$GT negative tumours.

**Table II** Relationship between $\gamma$GT activity and lymph node invasion

| | $\gamma GT^+$ | $\gamma GT^-$ |
|---|---|---|
| Absence of lymph node invasion | 9 | 17 |
| Presence of lymph node metastases | 27 | 17 |
| | $\chi^2=4.67$ | $P<0.05$ |

**Table III** Relationship between $\gamma$GT activity and pre- or postmenopausal status of the patients

| | $\gamma GT^+$ | $\gamma GT^-$ |
|---|---|---|
| Premenopause | 15 | 14 |
| Postmenopause | 21 | 20 |
| | not significant | |

### Discussion

A histoenzymatic analysis of human mammary tissue for $\gamma$GT activity has provided evidence for a difference between normal and benign tissue on the one hand and malignant tumours on the other.

**Table IV** Relationship between $\gamma$GT activity and oestradiol receptors (ER) and progesterone receptors (PGR) in carcinomas

| | $\gamma GT^+$ | $\gamma GT^-$ |
|---|---|---|
| $ER^+$ | 22 | 28 |
| $ER^-$ | 14 | 6 |
| | $\chi^2=3.28$ | $P\simeq 0.05$ |
| $PGR^+$ | 23 | 24 |
| $PGR^-$ | 13 | 10 |
| | not significant | |

Indeed, as previously stated, the staining pattern of normal and benign tissue was similar from one sample to the next whereas it was heterogeneous in carcinomas. Further, the distribution of enzyme activity was similar in normal and benign tissue in that it was confined to the lumina of the ducts and lobules, and to the apical pole of epithelial cells with little or no staining of the cytoplasm. On the contrary, the cytoplasm of certain malignant carcinomas showed intense activity though the intensity varied from one tumour to the next. In other carcinomas activity was greatly reduced or almost negligible. This variation in $\gamma$GT activity in human mammary carcinomas had been previously reported by Levine *et al.* (1983) who noted a tendency for the more poorly differentiated carcinomas (grades 2 and 3) to have a weaker $\gamma$GT activity than grade 1 tumours. We could find no supportive evidence among our observations on 70 different tumours. In fact there were no obvious histopathological differences detectable by light microscopic examination between $\gamma GT^+$ and $\gamma GT^-$ tumours. Possible reasons for this discrepancy may be that the authors did not control the specificity of the $\gamma$GT reaction with serine-borate as we have done here and that they did not perform a statistical examination of their results. They also had a distribution of carcinomas between the 3 grades different from ours. The tumours used in this study are indeed biased in favour of grade 1 tumours according to the Bloom and Richardson classification. However they do not represent the overall tumour incidence from patients attending the breast cancer clinic. This incidence was 25% grade 1, 45% grade 2 and 30% grade 3, over the period of the study. The bias is due to the availability of biopsy material at surgery. The only correlations which were statistically significant with $\gamma$GT activity were lymph node invasion, and the absence of oestradiol receptors ($ER^-$). As regards the former, Nemoto *et al.* (1980) have shown that lymph node invasion is an unfavourable prognostic sign. As regards the latter, Knight *et al.* (1977) and

Rich *et al.* (1978) have also provided evidence showing that patients with $ER^-$ tumours have a less favourable prognosis than those with $ER^+$ tumours. At the moment though, this latter result should be interpreted with caution since the progesterone receptor content seems to be of greater prognostic value than that of ER (McGuire & Clark, 1983; Saez *et al.*, 1983). The functional significance of γGT in tumour cells cannot be determined from the results of the histoenzymatic analysis reported here. However the role of this enzyme in γ-glutamyl amino acid transport is well documented and recently Bridges and Meister (1985) have suggested that the transport of γ-glutamyl amino acids is dependent on intracellular glutathione levels. Further Osuji (1980) has also shown that γGT has two amino acid transporting sites. One can therefore speculate that the activity in benign tissue may be a reflection of normal γ-glutamyl amino acid transport whereas in carcinomas it may be a reflection of impairment in intracellular glutathione metabolism which has also been reported in some transformed cells (Meister & Anderson, 1983). In connection with γGT itself, circulating enzyme levels determined before mastectomy were elevated in only 3 patients whose tumours were γGT positive. Our inability to find a significant correlation between tumour γGT positivity and circulating γGT may be due to the fact that our histoenzymatic analysis on γGT positive tumours were performed on only 2 grade 3 tumours compared to 32 grade 1 and grade 2 tumours.

Based on the two above mentioned criteria *viz.* lymph node invasion, and $ER^-$ tumours, γGT positivity would appear to be an unfavourable prognostic sign. The real value of the results reported here will be tested only in a few years time when survival rates between the two groups can be compared.

Meanwhile, the simplicity and the rapidity of the histoenzymatic method properly controlled for enzyme specificity are two arguments in favour of performing routine γGT determinations in histological examinations of breast cancer.

We are indebted to Dr Simone Saez for helpful discussion and for hormone receptor determinations. We thank Mr Yves du Bois and Mr Alain Joubert for preparing the illustrations, and Ms. Josiane Colonozet for typing the manuscript.

### References

ALMERSJO, O., BENGMARK, S. & HAFSTROM, L. (1976). Liver metastases found by follow-up of patients operated on for colorectal cancer. *Cancer*, **37**, 1454.

BECK, P.R., BELFIELD, A., SPOONER, R.J., BLUMGART, L.H. & WOOD, C.B. (1979). Serum enzymes in colorectal cancer. *Cancer*, **43**, 1772.

BLOOM, H.J.G. & RICHARDSON, W.W. (1957). Histological grading and prognosis in breast cancer. *Br. J. Cancer*, **11**, 359.

BRIDGES, R.J. & MEISTER, A. (1985). γ-glutamyl amino acids transport and conversion to 5-oxoproline in the kidney. *J. Biol. Chem.*, **260**, 7304.

CAMERON, R., KELLEN, J., KOLIN, A., MALKIN, A. & FARBER, E. (1978). γ-glutamyltransferase in putative premalignant liver cell populations during hepatocarcinogenesis. *Cancer Res.*, **38**, 823.

COOPER, E.H., TURNER, R., STEELE, L., NEVILLE, A.M. & MACKAY, A.M. (1975). The contribution of serum enzymes and carcinoembryonic antigen to the early diagnosis of metastatic colorectal cancer. *Br. J. Cancer*, **31**, 111.

DE YOUNG, L.M., RICHARDS, W.L., BONZELET, W., TSAI, L.L. & BOUTWELL, R.K. (1978). Localization and significance of γ-glutamyltranspeptidase in normal and neoplastic mouse skin. *Cancer Res.*, **38**, 3697.

EORTC Breast Cancer Cooperative Group (1973). Standards for the assessment of oestrogen receptors in human breast cancer. *Eur. J. Cancer*, **9**, 379.

FIALA, S., MOHINDRU, A., KETTERING, W.G., FIALA, A.E. & MORRIS, H.P. (1976). Glutathione and γ-glutamyltranspeptidase in rat liver during chemical carcinogenesis. *J. Natl Cancer Inst.*, **57**, 591.

HARADA, M., OKABE, K., SHIBATA, K., MASUDA, H., MIYOTA, K. & ENOMOTO, M. (1976). Histochemical demonstration of increased activity of γ-glutamyl-transpeptidase in rat liver during hepatocarcinogenesis. *Acta Histochem. Cytochem.*, **9**, 168.

HAWKINS, R.A., ROBERTS, M.M. & FORREST, A.P.M. (1980). Oestrogen receptors and breast cancer: current status. *Br. J. Surg.*, **67**, 153.

HIROTA, N. & WILLIAMS, G.M. (1979). The sensitivity and heterogeneity of histochemical markers for altered foci involved in liver carcinogenesis. *Am. J. Pathol.*, **95**, 317.

HORWITZ, K.B. & McGUIRE, W.L. (1975). Specific progesterone receptors in human breast cancer. *Steroids*, **25**, 497.

JAKEN, S. & MASON, M. (1978). Differences in the isoelectric focusing patterns of gamma-glutamyltrans-peptidase from normal and cancerous rat mammary tissue. *Proc. Natl. Acad. Sci.*, **75**, 1750.

KLEIN-SZANTO, A.J.P., NELSON, K.G., SHAH, Y. & SLAGA, T.J. (1983). Simultaneous appearance of keratin modifications and γ-glutamyltransferase activity as indicators of tumor progression in mouse skin papillomas. *J. Natl., Cancer Inst.*, **70**, 161.

KNIGHT, W.A., LIVINGSTON, R.B., GREGORY, E.J. & McGUIRE, W.L. (1977). Estrogen receptor as an independent prognostic factor for early recurrence in breast cancer. *Cancer Res.*, **37**, 4669.

LEVINE, S.E., BUDWITT, D.A., MICHALOPOULOS, G.K., GEORGIADE, G.S. & McCARTY, K.S. (1983). γ-glutamyltranspeptidase activity in benign and malignant human mammary epithelial lesions. Histochemical evaluation. *Arch. Pathol. Lab. Med.*, **107**, 423.

MARATHE, G.V., NASH, B., HASCHEMEYER, R.H. & TATE, S.S. (1979). Ultrastructural localization of γ-glutamyltranspeptidase in rat kidney and jejunum. *FEBS Lett.*, **107**, 436.

McGUIRE, W.L. & CLARK, G.M. (1983). Progesterone receptors and human breast cancer. *Eur. J. Cancer Clin. Oncol.*, **19**, 1681.

MEISTER, A. & ANDERSON, M.E. (1983). Glutathione. *Ann. Rev. Biochem.*, **52**, 711.

MUNJAL, D., CHAWLA, P.L., LOKICH, J.J. & ZAMCHECK, N. (1976). Carcinoembryonic antigen and Phosphohexose isomerase, γ-glutamyltranspeptidase and lactate deshydrogenase levels in patients with and without liver metastases. *Cancer*, **37**, 1800.

NEMOTO, T., VANA, J., BEDWANI, R.N., BAKER, H.W., McGREGOR, F.H. & MURPHY, G.P. (1980). Management and survival of female breast cancer: Results of a national survey by the American College of Surgeons. *Cancer Res.*, **45**, 2917.

ORGANISATION MONDIALE DE LA SANTE (1981). Classification internationale des tumeurs. Tumeurs du Sein. 2ème édition. Genève, O.M.S.

OSUJI, G.O. (1980). The kinetics of the γ-glutamyl cycle mediated uptake of amino acids. Considerations explaining the bifurcation of the γ-glutamyl cycle. *FEBS Lett.*, **110**, 192.

POCIUS, P.A., BAUMRUCKER, C.R., McNAMARA, J.P. & BAUMAN, D.E. (1980). γ-glutamyltranspeptidase in rat mammary tissue. Activity during lactogenesis and regulation by prolactin. *Biochem. J.*, **188**, 565.

PUENTE, J., VARAS, M.A., BECKHAUS, G. & SAPAG-HAGAR, M. (1979). γ-glutamyltranspeptidase activity and cyclic AMP levels in rat liver and mammary gland during the lactogenic cycle and in the oestradiol-progesterone pseudo-induced pregnancy. *FEBS Lett.*, **99**, 215.

RANSON, J.H., ADAMS, P. & LOCALIO, S.A. (1973). Preoperative assessment for hepatic metastases in carcinoma of the colon and rectum. *Surg. Gynecol. Obstet.*, **137**, 435.

RICH, M.A., FURMANSKI, P. & BROOKS, S.C. (1978). Prognostic value of estrogen receptor determinations in patients with breast cancer. *Cancer Res.*, **38**, 4296.

RUTENBURG, A.M., KIM, H., FISCHBEIN, J.W., HANKER, J.S., WASSERKRUG, H.L. & SELIGMAN, A.M. (1969). Histochemical and ultrastructural demonstration of γ-glutamyltranspeptidase activity. *J. Histochem. Cytochem.*, **17**, 517.

SAEZ, S., CHEIX, F. & ASSELAIN, B. (1983). Prognostic value of estrogen and progesterone receptors in primary breast cancer. *Breast Cancer Res. Treat.*, **3**, 345.

TATE, S.S. & MEISTER, A. (1978). Serine-borate complex as a transition-state inhibitor of γ-glutamyltranspeptidase. *Proc. Natl Acad. Sci.*, **75**, 4806.